# Cell-Cycle Analysis of Fission Yeast Cells by Flow Cytometry

**DOI:** 10.1371/journal.pone.0017175

**Published:** 2011-02-28

**Authors:** Jon Halvor Jonsrud Knutsen, Idun Dale Rein, Christiane Rothe, Trond Stokke, Beáta Grallert, Erik Boye

**Affiliations:** 1 Department of Cell Biology, Institute for Cancer Research, Oslo University Hospital, Oslo, Norway; 2 Department of Radiation Biology, Institute for Cancer Research, Oslo University Hospital, Oslo, Norway; 3 Institute for Molecular Bioscience, University of Oslo, Oslo, Norway; University College London, United Kingdom

## Abstract

The cell cycle of the fission yeast, *Schizosaccharomyces pombe*, does not easily lend itself to analysis by flow cytometry, mainly because cells in G_1_ and G_2_ phase contain the same amount of DNA. This occurs because fission yeast cells under standard growth conditions do not complete cytokinesis until after G_1_ phase. We have devised a flow cytometric method exploiting the fact that cells in G_1_ phase contain two nuclei, whereas cells in G_2_ are mononuclear. Measurements of the width as well as the total area of the DNA-associated fluorescence signal allows the discrimination between cells in G_1_ and in G_2_ phase and the cell-cycle progression of fission yeast can be followed in detail by flow cytometry. Furthermore, we show how this method can be used to monitor the timing of cell entry into anaphase. Fission yeast cells tend to form multimers, which represents another problem of flow cytometry-based cell-cycle analysis. Here we present a method employing light-scatter measurements to enable the exclusion of cell doublets, thereby further improving the analysis of fission yeast cells by flow cytometry.

## Introduction

**Figure 1:**
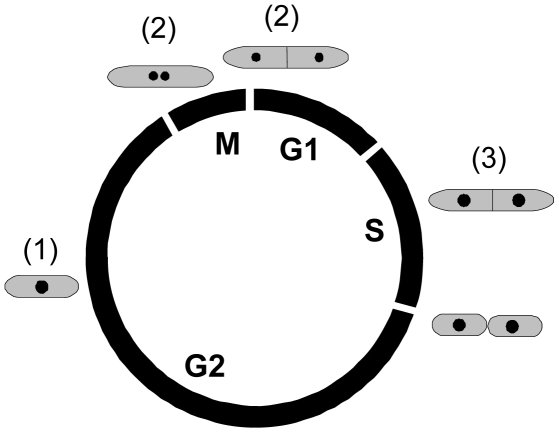
Schematic representation of the *S. pombe* cell cycle. The circle indicates the relative positions and durations of the different cell-cycle phases. The bodies outside the circle indicate the morphology of cells at the different phases and the numbers in parentheses show the subpopulations that they belong to (see Figure 2). The nuclei are indicated by dark spots.

The fission yeast, *Schizosaccharomyces pombe*, is a popular model system, amenable to classic and molecular genetic analysis as well as biochemical and physiological studies [1]. The cell-cycle progression of fission yeast can be measured by flow cytometry, which is a powerful method to analyse many aspects of cell-cycle regulation for most organisms. However, because of two special features analysis of fission yeast cell growth by flow cytometry is not straightforward: First, under standard laboratory conditions the cytokinesis of fission yeast cells occurs at the end of S phase and for that reason cells in G_1_ and S phase are binuclear (Fig. 1). Cells in G_1_ phase contain two nuclei, each with a single, complete genome (termed 1C DNA) and these cells contain the same total amount of DNA (2C) as cells in G_2_ phase, which harbor their DNA in a single nucleus. Therefore, the discrimination of G_1_ cells from G_2_ cells is not straightforward by simple measurements of the cellular DNA content in a flow cytometer. Second, the fission yeast cells tend to form multimers by sticking together, thereby perturbing flow cytometric analyses of single-cell behaviour and of cell-cycle kinetics. Here we show how these problems can be solved. The methods presented are technically simple and available to most flow cytometry users. We also give examples of useful applications of the novel methods.

## Results and Discussion

**Figure 2:**
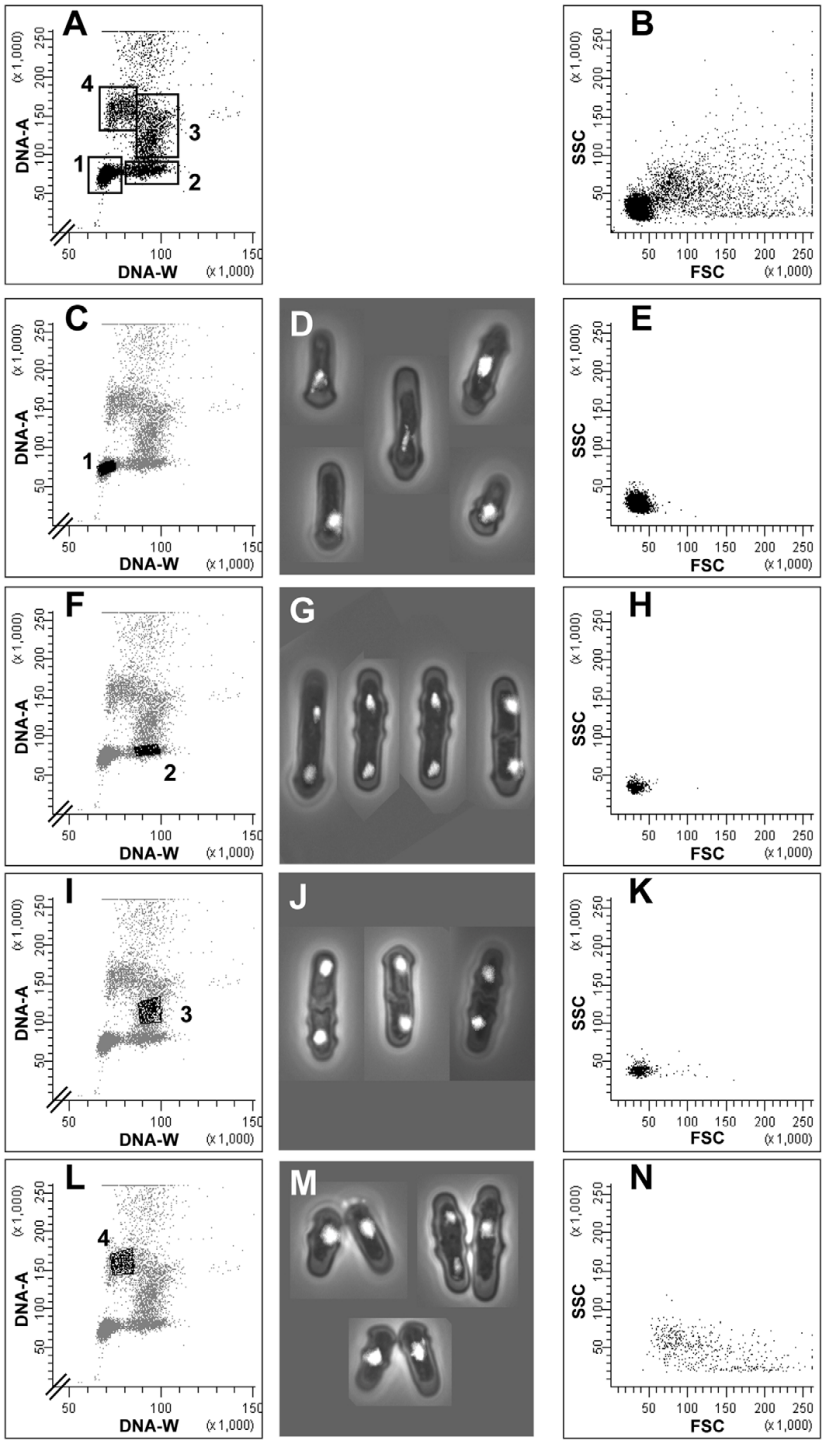
Analysis of exponentially growing cells. Flow cytometric and microscopic analyses of wild-type *S. pombe* cells in an exponentially growing culture. First column: cytograms showing DNA-W versus DNA-A; second column: microscopy images of the sorted cells; third column: cytograms showing FSC versus SSC. The four major subpopulations are indicated in panel A and they were analyzed individually by sorting, as shown in panels C, F, I and L.

The major problem in analyzing *Schzosaccharomyces pombe* cells and their cell cycle by flow cytometry is that they contain the same amount of DNA in G_1_ and in G_2_ phase. Here we have exploited the physical shape of fission yeast cells, namely that they are rod-shaped. Such cells are, due to hydrodynamic focusing in flow cytometers, oriented parallel to the laminar flow when passing through the excitation focus. A cell containing two separate nuclei passing sequentially through the excitation focus will give a longer-lasting DNA-associated fluorescence signal than a cell with one nucleus, and in particular with a narrow and well focused field of excitation. The reason is that fluorescently labeled DNA will remain in the excitation focus for a longer period of time when there are two separated nuclei than when there is only one nucleus in the cell. Therefore, the DNA signal (DNA-W) is expected to last longer for a binuclear than for a uninuclear cell. In contrast, the total area of the DNA signal (DNA-A), reflecting total DNA content of the particle passing the excitation focus, should be unaffected by nuclear distribution. Experimentally, the two-parametric DNA-W/DNA-A cytograms for exponentially growing cells revealed four main subpopulations (Figure 2A). We isolated the different populations by fluorescence-activated cell sorting and analyzed them by microscopy for identification. The results showed clearly that we could separate G_2_ cells with a single nucleus and a 2C DNA content (subpopulation 1) from cells in G_1_ phase or late in mitosis that also have a 2C DNA content (subpopulation 2; Figure 2C, D, F, G). Furthermore, cells in S phase, with a DNA content between 2C and 4C, could be clearly identified (subpopulation 3; Figure 2 I, J). In addition, forward light scatter (FSC) and side-scatter (SSC) were measured for all particles. Cell doublets (subpopulation 4; Figure 2L) were found with a very broad distribution in the FSC/SSC cytograms, but well outside the tight distribution observed for cell singlets (compare single cells in Figure 2E, H and K with cell doublets in Figure 2N). The reason for the good separation between single cells and cell doublets in the FSC/SSC cytogram is that there is little or no increase in the forward scattering as cells grow in length, which results in almost the same FSC/SSC values for single cells. We conclude that single cells and cell doublets can be distinguished from one another by this method. This will greatly improve and extend the application of flow cytometry to analyze the cell cycle of fission yeast cells.

This lack of sensitivity to cell length is not due to so-called “anomalous diffraction”[2], since this latter phenomenon varies with the wavelength of the exciting light. We find that the lack of increase in light scattering with cell length was independent of whether excitation was performed at 405, 488, 561, or 640 nm (data not shown). The blocking bar, employed in all flow cytometers to exclude direct laser exposure and reflection into the detectors, also excludes light scattered below a certain critical angle. It is likely that, as the cells grow longer, the increased light scattering due to the increased long axis of the cells will fall below the critical angle and will thus not contribute significantly to the measured scattering signal.

**Figure 3:**
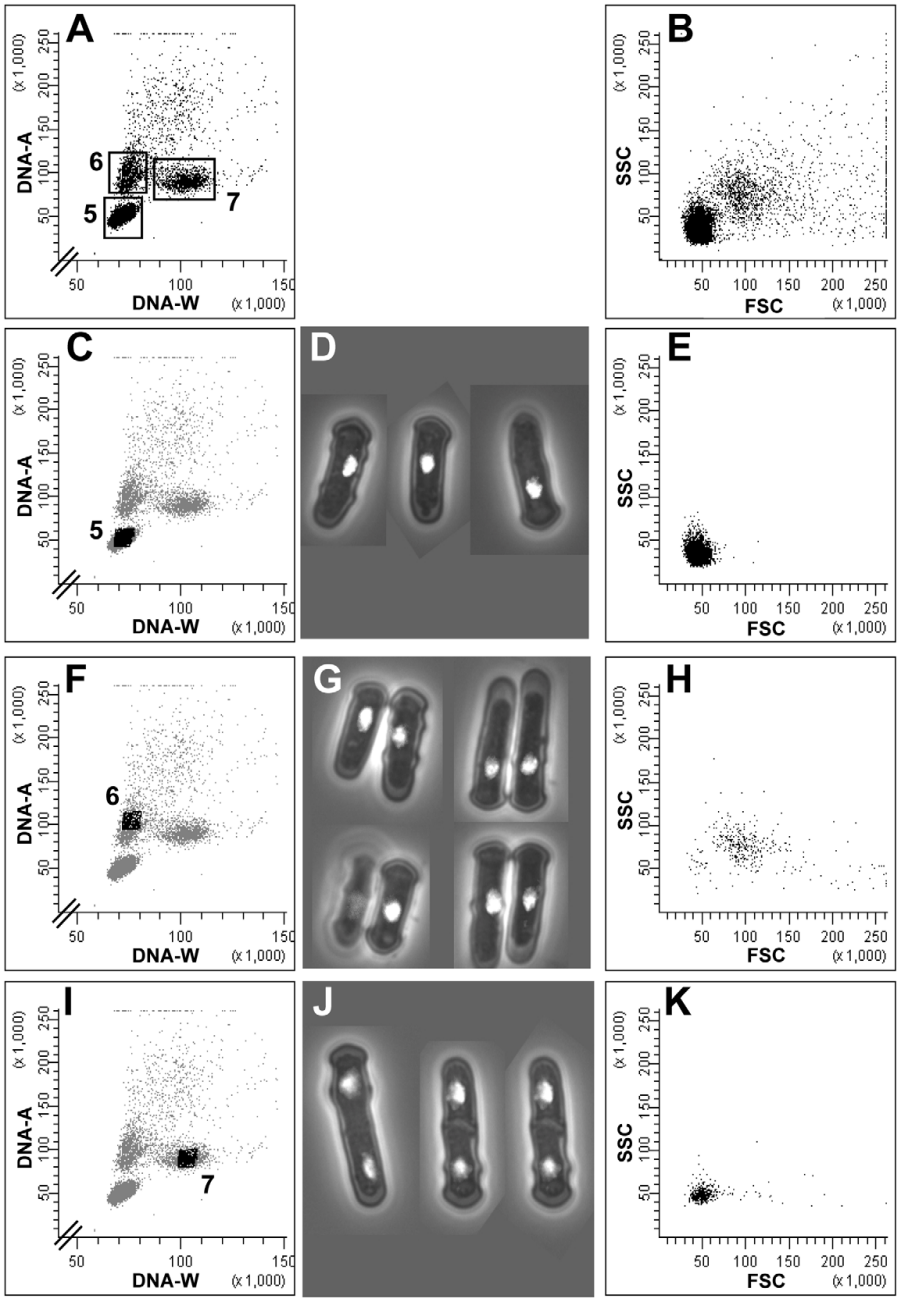
Analysis of cells arrested in G_1_ phase. Flow cytometric and microscopic analyses of *S. pombe* cells in cells arrested in G_1_ phase. First column: cytograms showing DNA-W versus DNA-A; second column: microscopy images of the sorted cells; third column: cytograms showing FSC versus SSC. The three major subpopulations are indicated in panel A and they were analyzed individually by sorting, as shown in panels C, F and I.

Similar analyses were performed on cells arrested in G_1_ phase by incubating a population of a *cdc10ts* strain at the restrictive temperature. The cells in such a culture formed three main subpopulations (Figure 3A). Subpopulation 5 represented single cells with one nucleus, a 1C DNA content and low FSC/SSC values (Figure 3C, D, E), i.e. cells arrested in G_1_ phase that had performed cytokinesis during the arrest. The vast majority of the cells were found in this population and could constitute the basis for synchronized release into the cell cycle from G_1_ phase. Subpopulation 6 consisted almost exclusively of cell doublets of two cells in G_1_ phase (Figure 3G). Consistently, the FSC/SSC values for these cells were much higher (Figure 3H) than for the cells with 1C DNA content (also cells in G_1_ phase) in subpopulation 5 (Figure 3E). However, some cells in subpopulation 6 were found with low FSC/SSC, probably representing G_2_ cells still present in the sample, i.e. corresponding to subpopulation 1 in Figure 2C. Subpopulation 7 consisted of particles with 2C DNA content and a larger DNA-W signal than for particles in subpopulations 5 and 6, represented single cells with two nuclei and were found in the lower part of the FSC/SSC plot (Figure 3I, J, K). This characterizes cells in late mitosis or G_1_ phase and this analysis identified cells that had not gone through cytokinesis during the temperature shift. This subpopulation is equivalent to Subpopulation 2 in the analysis of exponentially growing cells (Figure 2A). These results confirm and extend the conclusions above, namely that measurements of DNA-A and DNA-W by flow cytometry allow discrimination between cells in G_1_ and G_2_ phase. Moreover, the method can be used to monitor the quality of a method of synchronization, since the subpopulations could be identified.

**Figure 4:**
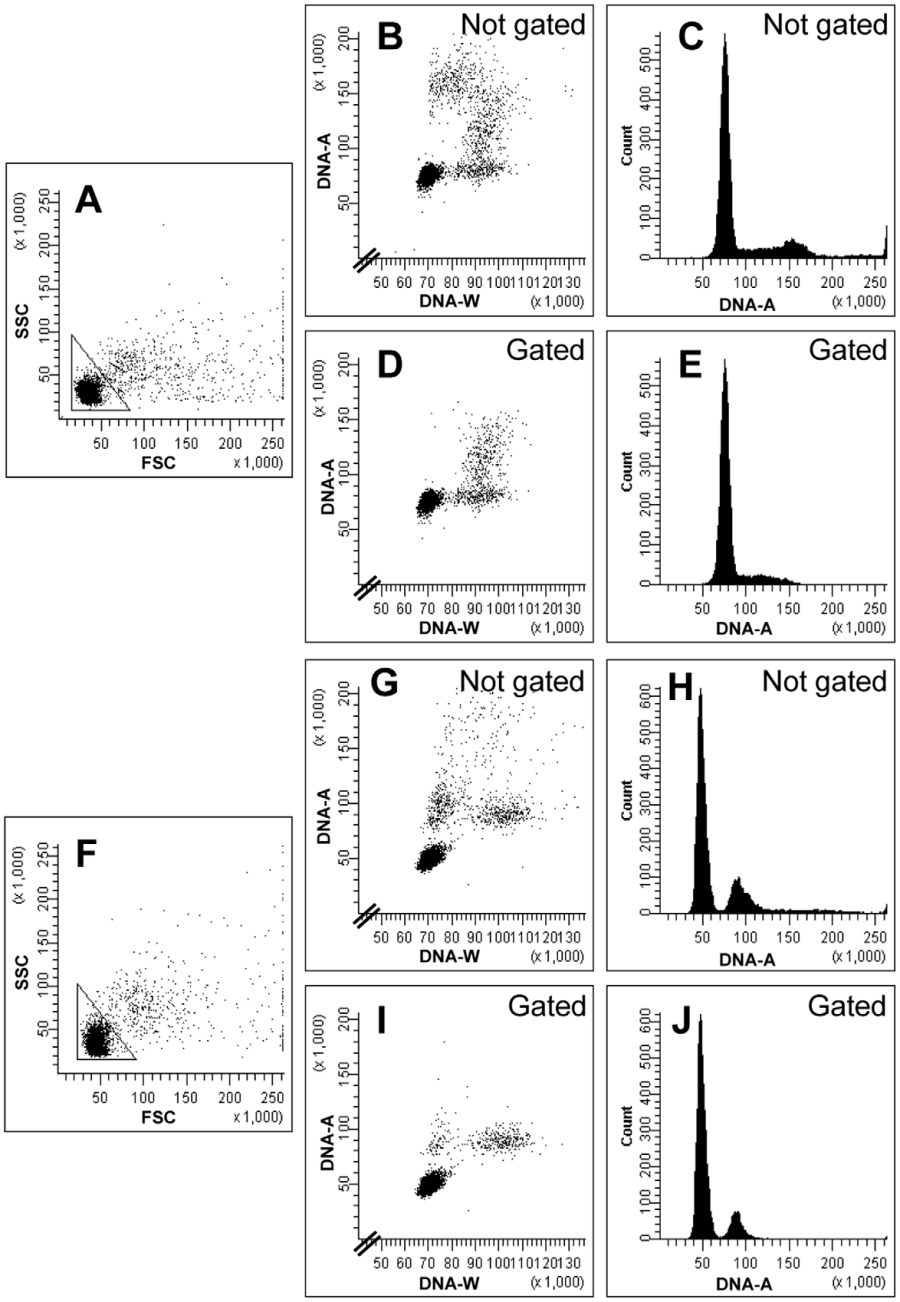
The importance of light-scatter gating. Flow cytometric analyses of a culture of exponentially growing *S. pombe* cells (top half) and cells arrested in G_1_ phase (bottom). The figure displays two-parametric light scatter cytograms (A, F), two-parametric DNA cytograms (B, D, G, I) and DNA histograms (C, E, H, J). Shown are the entire populations (A, B, C and F, G, H) and the remaining cells when gating has been introduced (D, E and I, J), thus removing cell doublets. The gates are shown in panels A and F.

The potential and power of the method to exclude cell doublets and to separate mononuclear from binuclear cells is illustrated in Figure 4, where we have analyzed an asynchronous (panels A–E) and a G_1_-synchronized (panels F–J) population of cells without (panels B, C, G, H) and with gating (panels D, E, I, J) on FSC and SSC. The reduction in the numbers of cells with 4C DNA for the asynchronous population (panel C versus E) and with 2C DNA for the synchronized population (panel H versus J) clearly shows that gating on this region in the FSC vs. SSC cytogram is necessary to obtain reliable DNA histograms of single cells and therefore to arrive at the correct cell-cycle distribution. Whereas the presence of cells with 4C DNA content in the ungated histogram (panel C) could suggest the presence of cells in G_2_ phase that had not yet passed through cytokinesis, gating on light scattering revealed that these cells were in fact cell doublets (panel E). Therefore, cytokinesis occurred before or at the S/G_2_ transition rather than in G_2_ phase. Sonication of the sample reduced the number of cell doublets, but not sufficiently to replace gating on FSC/SSC (Supplementary Figure S1). The true DNA content distributions of mononuclear and binuclear cells were obtained after additional gating with low and high DNA-W, respectively, as described above. After renormalization according to the percentage of cells with low and high DNA-W, the fractions of cells in the different compartments were obtained (Table 1). From these fractions, and the cell cycle time, it is possible to calculate the length of the G_2_, G_1_ and S phases. Table 1 shows that the population of cells with 2C DNA content in the G_1_- arrested culture contain cells in G_1_ phase that have not divided, but also a small fraction of cells in G_2_ phase.

**Table 1:** Quantification of cells in different cell-cycle phases.

Cell culture	Gating		1C DNA	1-2C DNA	2C DNA	2-4C DNA	4C DNA
Asynchronous	Ungated		0	0	72	15	13
	Gated on FSC/SSC and	Low DNA-W	0	0	77 (S1, i.e. G_2_)	0	0
		High DNA-W			9 (S2, i.e. 2xG_1_)	14 (S3, i.e. 2xS)	0
G_1_-arrested	Ungated		63	0	37	0	0
	Gated on FSC/SSC and	Low DNA-W	87 (S5, i.e. G_1_)	0	3 (S6, i.e. G_2_)	0	0
		High DNA-W			10 (S7,i.e.2xG_1_)	0	0

The numbers show percent cells with different DNA contents, emerging from the data shown in Figure 4C (line 1), Figure 4D (lines 2,3), Figure 4H (line 4) and Figure 4J (lines 5,6). The S numbers in parenthesis refer to the subpopulations defined in Figures 2 and 3. Gating was first performed with respect to FSC/SSC to exclude cell doublets. Thereafter, the DNA content distributions of cells with low DNA-W, i.e. mononuclear cells, and with high DNA-W (binuclear cells) were analyzed separately by simulations. The data were further renormalized according to the fractions of cells with low and high DNA-W.

**Figure 5:**
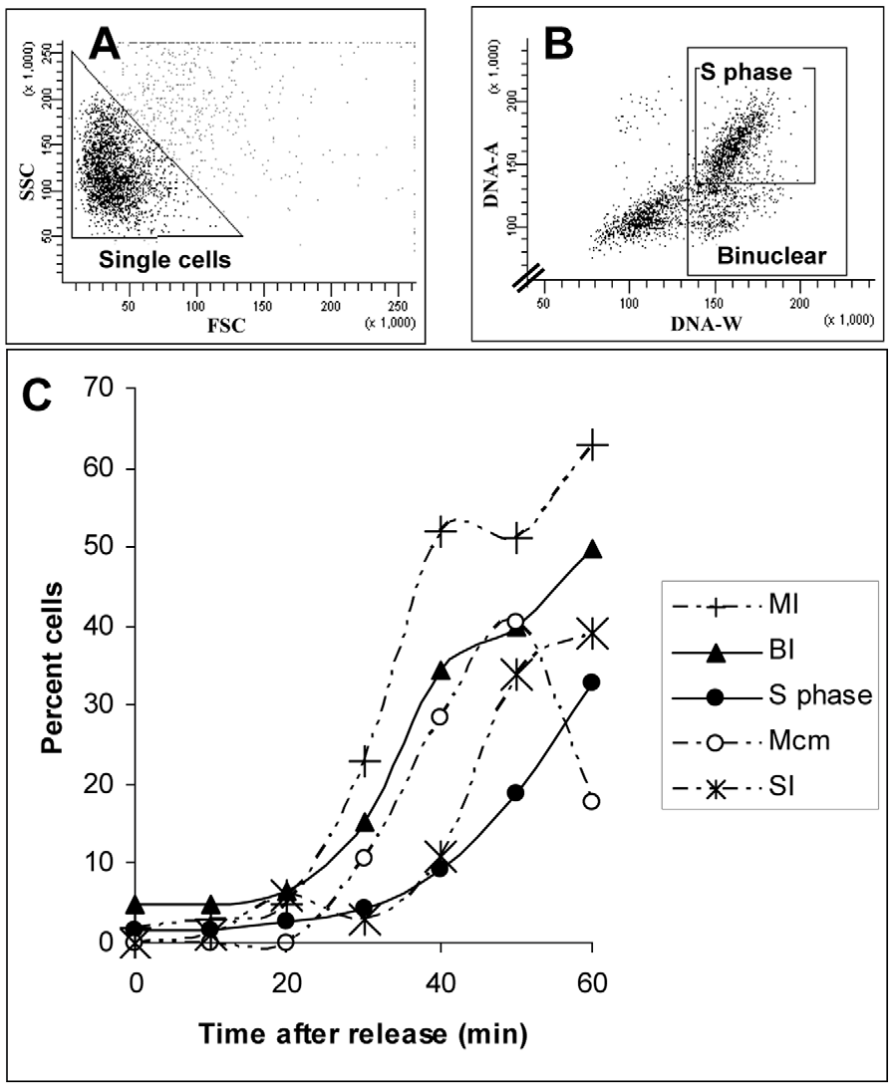
Monitoring cell-cycle progression through mitosis. Cell-cycle kinetics of a culture arrested in G_2_ phase and released into the cell cycle for 60 min. Cell doublets were eliminated by employing the gate shown in panel A, resulting in the DNA cytogram shown in panel B. Indicated are the positions of the binuclear cells and of cells in S phase. Panel C: quantification of the MI, BI, SI, the frequency of S-phase cells and of cells with chromatin-bound Mcms as a function of time after release. Results are shown for one representative experiment where all parameters were measured on the very same cell sample.

The ability to separate mononuclear from binuclear cells was exploited to monitor, by flow cytometry, the passage of fission yeast cells through mitosis. The standard method to achieve this involves microscopic measurements of the frequency of septating cells, the septation index (SI), and/or the frequency of cells that have passed into or through mitosis, the mitotic index (MI). Here we have employed flow cytometry to measure the frequency of binuclear cells (the binuclear index, BI) and compared the BI, the MI and the SI for one and the same cell culture synchronized in G_2_ phase and released into the cell cycle. After applying the gate to eliminate cell doublets (Figure 5A) we identified binuclear cells (Figure 5B), i.e. the cells found in subpopulations 2 and 3 of Figure 2A. The time and kinetics of rise in BI corresponded with the rise in MI and SI, but occurred in-between the MI and the SI (Figure 5C). We conclude that flow cytometric measurement of the BI is a convenient and simple method to estimate the time of mitotic entry.

Previous approaches to monitor entry into S phase include the measurement of the SI or of incorporation of externally supplied bromodeoxyuridine (BrdU) [3], [4]. The first method may be misleading, since there is no obligatory link between septum formation and S-phase entry and in many cases septation can finish well before S phase has started [5]. The second approach is tedious and requires extensive strain constructions because fission yeast cells do not take up BrdU and do not express thymidine kinase, which is required for BrdU incorporation into DNA. Here we have measured the frequency of S-phase cells by flow cytometry and found that this identification is easy and straightforward in a two-parametric analysis (Figure 5 A, B). For comparison, we have measured, in the same culture, the appearance of cells that contain Mcms bound to chromatin, an obligatory event before S-phase entry [4]. The frequency of cells with chromatin-bound Mcms rises slightly after the time of increase of the BI, in accordance with the known assembly of the pre-RC around the M/G1 transition [6]. As expected, the frequency of S-phase cells increases later than the increase seen in the MI, BI, SI and Mcm binding (Figure 5C). Thus, all five parameters show a timing of increase consistent with previous reports and we conclude that flow cytometry can be efficiently used to monitor mitotic entry in fission yeast cells. Furthermore, flow cytometry allows, in contrast to analyses by microscopy, the analysis of a large number of cells in a short time.

This work presents major advances in the analysis of fission yeast cells by flow cytometry. First, we have presented a method allowing the separation of binuclear from mononuclear cells. Second, another method presented facilitates the elimination of cell doublets; such doublets are formed at a significant frequency when the cells are fixed. These two methods will greatly improve single-cell analyses of fission yeast by flow cytometry and thereby allow a much more detailed, exact and sophisticated cell-cycle analysis. Furthermore, this application opens up novel possibilities to screen for fission yeast mutants with deficiencies in cell-cycle regulation, i.e. where the wanted phenotype involves arrest in a specific cell-cycle phase.

## Materials and Methods

### Yeast strains and cell handling

All the strains were derivatives of the *Schizosaccharomyces pombe* L972 h^-^ wild type strain [7]. The cells were grown exponentially in liquid Edinburgh Minimal Medium (EMM) to a cell concentration of 2-4×10^6^/ml (equivalent to an optical density at 595 nm of 0.1–0.2). To synchronize the cells in G_1_ or in G_2_ phase the temperature-sensitive *cdc10-M17* [8]or the *cdc25-22* [9] mutant, respectively, was used. The mutants were grown at the permissive temperature (25°C), shifted to 36°C for four hours before return to the permissive temperature.

### Preparation of cells for flow cytometry

The cells were handled and fixed for flow cytometry essentially as described earlier [10]. Briefly, about 2×10^6^ cells were pelleted at 16 000 x g for 1 min. The supernatant was removed and 1 ml of 70% cold ethanol was slowly added during vigorous mixing. Samples were stored at 4°C. For flow cytometry, 500 µl was transferred to a new tube and the ethanol was removed by centrifugation. The cell pellet was washed twice in 500 µl 20 mM EDTA, pH 8.0, before 0.1 mg/ml Ribonuclease A (Sigma-Aldrich) in 500 µl 20 mM EDTA, pH 8.0, was added and the samples were incubated for 3–16 hours at 36°C. Shortly before analysis, 500 µl of 20 mM EDTA, pH 8.0, and 2 µM Sytox Green (Invitrogen) were added. The samples were sonicated in 1.5 ml tubes in an ultrasonic water bath (VWR Ultrasonic Cleaner). Samples were stored on ice and protected from light before analysis.

### Flow cytometry

Stained cells were analyzed and sorted based on area (DNA-A) and pulse width (DNA-W) of the Sytox Green fluorescence signal, or on forward (FSC) and side (SSC) light scattering (area). Analysis was performed in an LSRII (Becton Dickinson, San Jose, CA, U.S.A.) with excitation at 488 nm. Cells were sorted in a FACS Digital Vantage SE flow cytometer (Becton Dickinson) with excitation at 488 nm. Sytox Green fluorescence was in both cytometers collected through a 530/30 emission filter.

### Microscopic analyses

Measurements of the formation of Mcm binding was performed by introducing the *mcm6*:GFP allele [6], [11] and to visualise the presence of the tagged protein in Triton-extracted cells by fluorescence microscopy. Established procedures were used to measure the SI after aniline blue staining [12] and MI after DAPI staining [13].

### Cell-cycle analyses of DNA histograms

The DNA contents of exponentially growing wild type cells and of *cdc10ts* cells arrested in G_1_ phase were measured by flow cytometry and the DNA histograms were analyzed by the FlowJo Software (Tree Star Inc, Ashland, Oregon, U.S.A.). The percentages of cells with different DNA contents were determined from the DNA histograms generated with and without gating.

## Supporting Information

Figure S1 **Sonication reduces the number of cell doublets.** The percentage of cell doublets remaining after sonication of flow cytometry samples prepared from fission yeast cultures in exponential growth. The mean and standard deviation of data from three independent experiments are shown. Sonication was performed in 1.5 mL tubes in an ultrasonic water bath (VWR Ultrasonic Cleaner, Radnor, PA) for the indicated times. Cells were counted electronically as single cells or doublets using the gating based on the FFS/SSC values as discussed in the text.
(TIF)
