# Supplementary figures and images for: Cell-Cycle Analysis of Fission Yeast Cells by Flow Cytometry
Source: PLoS One. 2011 Feb 28;6(2):e17175. doi: 10.1371/journal.pone.0017175 (PMC3046126; doi:10.1371/journal.pone.0017175)

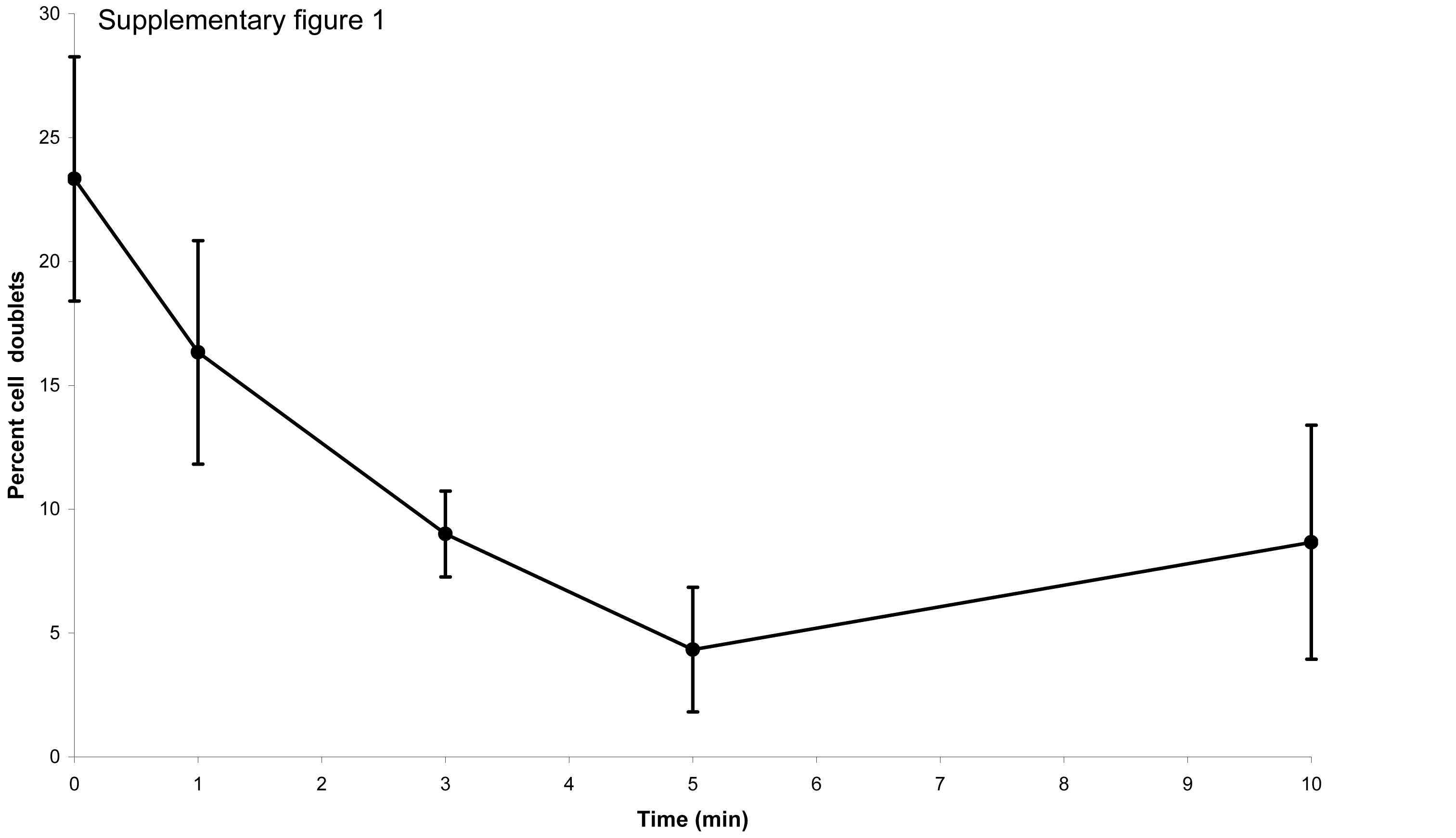

Supplement: Figure S1 — Sonication reduces the number of cell doublets. The percentage of cell doublets remaining after sonication of flow cytometry samples prepared from fission yeast cultures in exponential growth. The mean and standard deviation of data from three independent experiments are shown. Sonication was performed in 1.5 mL tubes in an ultrasonic water bath (VWR Ultrasonic Cleaner, Radnor, PA) for the indicated times. Cells were counted electronically as single cells or doublets using the gating based on the FFS/SSC values as discussed in the text. (TIF) [file pone.0017175.s001.tif]
